# Multinational prospective cohort study of rates and risk factors for ventilator-associated pneumonia over 24 years in 42 countries of Asia, Africa, Eastern Europe, Latin America, and the Middle East: Findings of the International Nosocomial Infection Control Consortium (INICC)

**DOI:** 10.1017/ash.2022.339

**Published:** 2023-01-09

**Authors:** Victor Daniel Rosenthal, Zhilin Jin, Ziad A. Memish, Camilla Rodrigues, Sheila Nainan Myatra, Mohit Kharbanda, Sandra Liliana Valderrama-Beltran, Yatin Mehta, Mohammad Abdellatif Daboor, Subhash Kumar Todi, Guadalupe Aguirre-Avalos, Ertugrul Guclu, Chin Seng Gan, Luisa Fernanda Jiménez Alvarez, Rajesh Chawla, Sona Hlinkova, Rajalakshmi Arjun, Hala Mounir Agha, Maria Adelia Zuniga Chavarria, Narangarav Davaadagva, Mat Nor Mohd Basri, Katherine Gomez, Daisy Aguilar De Moros, Chian-Wern Tai, Alejandro Sassoe Gonzalez, Lina Alejandra Aguilar Moreno, Kavita Sandhu, Jarosław Janc, Mary Cruz Aleman Bocanegra, Dincer Yildizdas, Yuliana Andrea Cano Medina, Maria Isabel Villegas Mota, Abeer Aly Omar, Wieslawa Duszynska, Souad BelKebir, Amani Ali El-Kholy, Safaa Abdulaziz Alkhawaja, George Horhat Florin, Eduardo Alexandrino Medeiros, Lili Tao, Nellie Tumu, May Gamar Elanbya, Reshma Dongol, Vesna Mioljević, Lul Raka, Lourdes Dueñas, Nilton Yhuri Carreazo, Tarek Dendane, Aamer Ikram, Souha S. Kanj, Michael M. Petrov, Asma Bouziri, Nguyen Viet Hung, Vladislav Belskiy, Naheed Elahi, María Marcela Bovera, Ruijie Yin

**Affiliations:** 1 Department of Public Health Sciences, University of Miami Miller School of Medicine, Miami, Florida, United States; 2 International Nosocomial Infection Control Consortium (INICC) Foundation, Miami, Florida, United States; 3 King Saud Medical City, Ministry of Health, Riyadh, the Kingdom of Saudi Arabia; 4 Pd Hinduja National Hospital and Medical Research Centre, Mumbai, India; 5 Tata Memorial Hospital, Homi Bhabha Nacional Institute, Mumbai, India; 6 Desun Hospital, Kolkata, India; 7 Pontificia Universidad Javeriana Hospital Universitario San Ignacio, Bogotá, Colombia; 8 Medanta the Medicity, Haryana, India; 9 King Hussein Cancer Center, Amman, Jordan; 10 Advanced Medicare Research Institute (AMRI) Hospitals, Kolkata, India; 11 Hospital Civil de Guadalajara Fray Antonio Alcalde. Centro Universitario de Ciencias de la Salud, Universidad de Guadalajara, Guadalajara, México; 12 Sakarya University Training and Research Hospital, Sakarya, Turkey; 13 University Malaya Medical Centre, Kuala Lumpur, Malaysia; 14 Clinica Universitaria Colombia, Bogotá, Colombia; 15 Indraprastha Apollo Hospitals, New Delhi, India; 16 Catholic University in Ruzomberok, Faculty of Health, Central Military Hospital Ruzomberok, Ruzomberok, Slovakia; 17 Kerala Institute of Medical Sciences and Health, Trivandrum, India; 18 Cairo University Specialized Pediatric Hospital, Cairo, Egypt; 19 Hospital Clínica Biblica, San José de Costa Rica, Costa Rica; 20 Intermed Hospital, Ulaanbaatar, Mongolia; 21 International Islamic University Malaysia, Kuantan Pahang, Malaysia; 22 Clinica Sebastián de Belalcázar, Cali, Colombia; 23 Hospital del Niño Dr José Renán Esquivel, Panamá, Panamá; 24 Universiti Kebangsaan Malaysia Specialist Children’s Hospital, Kuala Lumpur, Malaysia; 25 Hospital Regional de Alta Especialidad Ixtapaluca, Ixtapaluca, México; 26 Clinica Infantil Santa María del Lago, Bogotá, Colombia; 27 Max Super Specialty Hospital Saket Delhi, New Delhi, India; 28 Department of Anesthesiology and Intensive Therapy, 4th Clinical Military Hospital with Polyclinic, Wroclaw, Poland; 29 Hospital San José TecSalud, Monterrey, Nuevo Leon, Mexico; 30 Cukurova University. Balcali Hospital, Adana, Turkey; 31 Instituto Del Corazón De Bucaramanga Sede Bogotá, Bogotá, Colombia; 32 Instituto Nacional de Perinatología, México DF, México; 33 Infection Control Directorate. Ministry of Health, Kuwait City, Kuwait; 34 Department of Anesthesiology and Intensive Therapy, Wroclaw Medical University. Wroclaw, Poland; 35 An Najah National University Hospital, Nablus, Palestine; 36 Dar Alfouad Hospital, 6th of October City, 6th of October City, Egypt; 37 Salmaniya Medical Center, Manama, Bahrain; 38 University of Medicine and Pharmacy, Victor Babes Timisoara Emergency Clinical County Hospital Romania, Timisoara, Romania; 39 Hospital Sao Paulo, Universidade Federal de Sao Paulo, Sao Paulo, Brazil; 40 Zhongshan Hospital, Fudan University, Shanghai, China; 41 Port Moresby General Hospital, Port Moresby, Papua New Guinea; 42 National Infection control Program, Khartoum, Sudan; 43 Grande International Hospital, Kathmandu, Nepal; 44 Clinical center of Serbia, Belgrade, Serbia; 45 National Institute For Public Health, Prishtina, Kosovo; 46 Hospital Nacional de Niños Benjamin Bloom, San Salvador, El Salvador; 47 Universidad Peruana de Ciencias Aplicadas, Hospital de Emergencias Pediatricas, Lima, Peru; 48 Hôpital Ibn Sina, Rabat, Morocco; 49 National Institutes of Health, Islamabad, Pakistan; 50 American University Of Beirut Medical Center, Beirut, Lebanon; 51 St George University Hospital, Plovdiv, Bulgaria; 52 Hôpital d’enfants, Tunis, Tunisia; 53 Bach Mai Hospital, Hanoi, Vietnam; 54 Privolzhskiy District Medical Center, Nizhniy Novgorod, Russia; 55 Dubai Hospital, Dubai, United Arab Emirates; 56 Hospital De Los Valles, Ecuador

## Abstract

**Objective:**

Rates of ventilator-associated pneumonia (VAP) in low- and middle-income countries (LMIC) are several times above those of high-income countries. The objective of this study was to identify risk factors (RFs) for VAP cases in ICUs of LMICs.

**Design:**

Prospective cohort study.

**Setting:**

This study was conducted across 743 ICUs of 282 hospitals in 144 cities in 42 Asian, African, European, Latin American, and Middle Eastern countries.

**Participants:**

The study included patients admitted to ICUs across 24 years.

**Results:**

In total, 289,643 patients were followed during 1,951,405 patient days and acquired 8,236 VAPs. We analyzed 10 independent variables. Multiple logistic regression identified the following independent VAP RFs: male sex (adjusted odds ratio [aOR], 1.22; 95% confidence interval [CI], 1.16–1.28; *P* < .0001); longer length of stay (LOS), which increased the risk 7% per day (aOR, 1.07; 95% CI, 1.07–1.08; *P* < .0001); mechanical ventilation (MV) utilization ratio (aOR, 1.27; 95% CI, 1.23–1.31; *P* < .0001); continuous positive airway pressure (CPAP), which was associated with the highest risk (aOR, 13.38; 95% CI, 11.57–15.48; *P* < .0001); tracheostomy connected to a MV, which was associated with the next-highest risk (aOR, 8.31; 95% CI, 7.21–9.58; *P* < .0001); endotracheal tube connected to a MV (aOR, 6.76; 95% CI, 6.34–7.21; *P* < .0001); surgical hospitalization (aOR, 1.23; 95% CI, 1.17–1.29; *P* < .0001); admission to a public hospital (aOR, 1.59; 95% CI, 1.35-1.86; *P* < .0001); middle-income country (aOR, 1.22; 95% CI, 15–1.29; *P* < .0001); admission to an adult-oncology ICU, which was associated with the highest risk (aOR, 4.05; 95% CI, 3.22–5.09; *P* < .0001), admission to a neurologic ICU, which was associated with the next-highest risk (aOR, 2.48; 95% CI, 1.78–3.45; *P* < .0001); and admission to a respiratory ICU (aOR, 2.35; 95% CI, 1.79–3.07; *P* < .0001). Admission to a coronary ICU showed the lowest risk (aOR, 0.63; 95% CI, 0.51–0.77; *P* < .0001).

**Conclusions:**

Some identified VAP RFs are unlikely to change: sex, hospitalization type, ICU type, facility ownership, and country income level. Based on our results, we recommend focusing on strategies to reduce LOS, to reduce the MV utilization ratio, to limit CPAP use and implementing a set of evidence-based VAP prevention recommendations.

The International Nosocomial Infection Control Consortium (INICC) published international reports providing data on ventilator-associated pneumonia (VAP) and clinical outcomes of low- and middle-income countries (LMICs) in 2006,^1^ 2008,^2^ 2010,^3^ 2012,^4^ 2014,^5^ 2016,^6^ 2019,^7^ and 2021.^8^ Device utilization in INICC ICUs was comparable to that reported by the US Centers for Disease Control and Prevention National Healthcare Safety Network (CDC-NHSN) for ICUs, but INICC VAP rates were greater.^3^ According to the CDC-NHSN, the VAP rate in medical surgical ICUs and all other ICUs with ≤15 beds in United States is 1.1 VAP cases per 1,000 mechanical ventilator (MV) days.^9^ In the most recent international data for INICC ICUs, the pooled VAP rate was 10 times greater than those reported for CDC-NHSN ICUs (11.47 vs 1.1 per 1,000 ventilator days).^8^ In INICC reports, the crude mortality rate in ICU patients without healthcare-associated infection (HAI) is 17.12% (95% CI, 16.93–17.32); for those with VAP it is 42.32% (95% CI, 40.61–44.09); and for those with VAP plus CLABSI plus CAUTI it is 63.44% (95% CI, 55.99–71.60).^8^ A recent study demonstrated that VAP is an independent risk factor for mortality in a multiple logistic regression analysis (adjusted OR [aOR], 1.48; *P* < .0001).^10^

The appropriate interventions to prevent VAP in LMICs have yet to be analyzed thoroughly and data are very limited. It is necessary to develop more definitive approaches for VAP prevention for implementation in LMICs. Researchers have identified the following VAP risk factors (RFs): tracheostomy,^11,12^ length of stay (LOS),^13,14^ older age,^15^ trauma patients,^16^ postsurgical patients,^17^ burns patients,^17^ longer duration of surgery,^18^ history of smoking,^18^ low serum albumin concentration,^17^ high score on the American Society of Anesthesiologists (ASA) Physical Status Classification System,^17^ Acute Physiology and Chronic Health Evaluation (APACHE II) score >20,^14^ acute respiratory distress syndrome,^19^ lung injury,^19^ chronic obstructive pulmonary disease,^16^ upper respiratory tract colonization,^16^ sinusitis,^16^ PaO_2_:FiO_2_ ratio <200 mmHg,^14^ oropharyngeal colonization,^15^ biofilm on the surface and within lumen of the endotracheal tube,^16^ duration of mechanical ventilation (MV),^14,15^ frequent change in ventilator circuit,^16^ lack of use of heat and moist exchange humidifiers,^16^ supine position,^15,20^ frequent reintubation,^16^ enteral feeding,^16^ multiple central venous line insertions,^12^ presence of catheter-related infection,^14^ paralytic agents,^16^ previous use of broad-spectrum antibiotics,^13,15^ and patients transported out of an ICU.^16^

Additional epidemiological studies need to be conducted to achieve an understanding of VAP risk factors in LMICs. Currently, no study has analyzed multiple countries simultaneously to identify VAP RFs in ICUs, nor has any study been conducted prospectively with a standardized form over 24 years. Also, no study has analyzed any of the following variables and their association with VAP: income level of the country according to the World Bank; facility ownership; hospitalization type; and ICU type. And all of these factors are important in understanding the unique challenges in LMICs.

The objective of this study was to simultaneously analyze the following 10 variables to identify VAP RFs in LMICs: (1) age, (2) sex, (3) duration of MV, (4) MV utilization ratio as marker of severity of illness of patients, (5) LOS, (6) type of respiratory support, (7) type of hospitalization, (8) ICU type, (9) facility ownership, and (10) income level according to the World Bank.

## Methods

### Study population and design

This prospective observational cohort study included patients admitted to 743 ICUs of 282 hospitals in 144 cities in 42 Asian, African, European, Latin American, and Middle Eastern countries across 24 years between July 1, 1998, and February 12, 2022.

### Prospective cohort in ICUs and surveillance of HAIs

Each patient’s data were gathered at the time of ICU admission. An infection prevention professional (IPP) visited each patient’s bedside daily from the time of admission until discharge. This analysis prospectively included all adult and pediatric patients hospitalized in an ICU with or without HAI, and their data were gathered utilizing the INICC Surveillance Online System (ISOS).^21^ An IPP brings a tablet to each hospitalized patient’s bedside in the ICU, signs in to the ISOS, and simultaneously uploads patient data.^21^

Information provided at the time of admission includes setting (eg, nation, city, name of the hospital, and the ICU type) as well as information about the patient such as age, type of hospitalization, use of invasive devices (central line [CL], MV, urinary catheter [UC]), and presence of infection.^21^ Every day until the patient is discharged, an IPP uploads details regarding invasive devices (CL, MV, and/or UC) and positive cultures (blood, urine, and respiratory samples) for each patient.^21^

If the patient has signs or symptoms of infection, an infectious diseases specialist approaches the patient to determine the presence of an HAI (CLABSI, VAP, or CAUTI). According to the CDC-NHSN, an IPP looks at a patient’s signs and symptoms, cultures, radiographs, and other criteria that fulfill definitions of HAI.^22^

Over the 24 years of this study, all IPPs of all participant hospitals have been applying the current and updated CDC definitions of HAI. That is, whenever the CDC updated their definitions, our IPPs began using the new updated definitions.

When IPPs upload the results of a culture to the ISOS, the ISOS immediately displays a message and directs the IPP to an online module of the ISOS where the IPP can check all the CDC-NHSN criteria to determine the presence of a HAI and the type of HAI (CLABSI, VAP, or CAUTI).^21^

Daily device utilization checks are performed by ISOS. When a bias in patient days or device use is detected from admission to discharge, the ISOS notifies an IPP. The ISOS data may show that the patient has been hospitalized in the ICU without any devices in place, most likely because the IPP forgot to upload the use of devices or forgot to upload the discharge of the patient. If the ISOS detects the lack of use of any kind of device on any given day, it sends a message to the IPP to upload missing devices or upload the discharge of the patient. In other words, the ISOS asks the IPP to investigate why a patient in an ICU does not have any devices in place.^21^ This approach significantly reduces biases associated with device utilization, patient days, and discharge conditions.^21^

Patients with missing data were excluded from this study. The institutional review boards of the participating hospitals approved this study. Patient and hospital identities have been excluded for confidentiality.

### INICC surveillance online system

Standard CDC-NSHN methodologies state that HAI denominators are device days gathered from all patients as pooled data, without mentioning the characteristics of particular patients or the quantity of device days associated with particular patients.^22^ INICC HAI surveillance is carried out through an online platform, the ISOS, which includes CDC-NHSN criteria and methods.^22^

Additionally, ISOS includes the gathering of patient-specific information on all patients, including those with and those without HAI, with a several variables per patient.^21^ The ability to match data from all patients admitted to ICUs by different variables allows for the estimation of the VAP RFs. The CDC-NHSN criteria and methods are used in the data uploaded to ISOS to identify HAIs, to estimate HAI rates, and to determine device utilization ratios.^22^

### Validation of diagnosis of healthcare-associated infections

Validation of an HAI is a unique feature of the ISOS and is considered essential for maximizing the sensitivity and accuracy of surveillance data. Each HAI reported by an IPP is validated, that is, scrutinized to ascertain that criteria are fulfilled to justify its recording as an HAI. All necessary corrections and additions are indicated with a clear red sign on the screen. The validation process also includes the scrutiny of data reported for putatively uninfected patients to permit detection of unreported but true HAI. To accomplish this, should the ISOS suspect an HAI when the IPP uploads a culture to the ISOS but does not confirm an HAI (based on the uploaded culture, the date that the culture was taken, and the result of the culture), the ISOS automatic validation system sends an online message to the IPP requesting a check of the CDC-NHSN criteria for that putative HAI. Also, the ISOS sends a Excel (.xls) file (Microsoft, Redmond, WA) to the IPP every month with a list of biases regarding HAIs that have not been confirmed.^21^

### Study definitions

Ventilator was defined as any device used to support, assist, or control respiration through the application of positive pressure to the airway when delivered via an artificial airway, specifically an oral or nasal endotracheal or tracheostomy tube. Definitions of VAP used during surveillance were those published by the CDC in 1991^23^ and included all subsequent updates through 2022.^24^ VAP was defined as pneumonia in which the patient had been on MV for >2 consecutive calendar days on the date of the event, with the day of ventilator placement being day 1, and the ventilator had been in place on the date of the event or the day before.^24^

Clinical pneumonia was defined as 2 or more serial chest-imaging results with at least 1 of the following: new and persistent or progressive and persistent, infiltrate, consolidation, cavitation, pneumatoceles (in infants aged ≤1 year). In addition, for any patient, at least 1 of the following must be present: fever, leukopenia or leukocytosis, or altered mental status with no other recognized cause (only in adults aged ≥70 years). Also, at least 2 of the following must be present: new onset of purulent sputum or change in character of sputum, increased respiratory secretions, or increased suctioning requirements; new-onset or worsening cough, dyspnea, or tachypnea; rales or bronchial breath sounds; worsening gas exchange; increased oxygen requirements; and/or increased ventilator demand.^24^

Pneumonia with common bacterial or filamentous fungal pathogens and specific laboratory findings was defined as 2 or more serial chest imaging test results with at least 1 of the following: new and persistent or progressive and persistent infiltrate; consolidation; cavitation; pneumatoceles (in infants aged ≤1 year). Also, at least 1 of the following must be present: fever, leukopenia or leukocytosis, or altered mental status with no other recognized cause (only in adults aged ≥70 years). In addition, for any patient, at least 1 of the following must be present: new onset of purulent sputum, change in character of sputum, or increased respiratory secretions, or increased suctioning requirements; new onset or worsening cough, dyspnea, or tachypnea; rales or bronchial breath sounds; worsening gas exchange; increased oxygen requirements; or increased ventilator demand. In addition, at least 1 of the following must be present: organism identified from blood; organism identified from pleural fluid; positive quantitative culture or corresponding semiquantitative culture result from minimally contaminated LRT specimen; ≥5% BAL-obtained cells contain intracellular bacteria on direct microscopic exam; positive quantitative culture or corresponding semiquantitative culture result of lung tissue; or histopathologic exam showing evidence of pneumonia.^24^

World Bank country classifications were defined in 4 income groups: low income, lower–middle income, upper–middle income, and high income. These classifications are based on gross national income (GNI) per capita expressed in current US dollars (USD). Low-income countries are those countries with a GNI <1,045 USD. Lower–middle income countries are those with a GNI between 1,046 and 4,095 USD. Upper–middle-income countries are those with a GNI between 4,096 and 12,695 USD. High-income countries are those with a GNI >12,695 USD.^25^

Device utilization was calculated as the ratio of device days to patient days for each location type. As such, the device utilization of a location measures the use of invasive devices and constitutes an extrinsic RF for HAI. Device utilization may also serve as a marker for the severity of illness of patients (ie, severely ill patients are more likely to require an invasive device), which is an intrinsic RF for infection.^26^

Facility and institution ownership type were defined as follows: publicly owned facilities owned or controlled by a governmental unit or another public corporation (where control is defined as the ability to determine the general corporate policy); not-for-profit privately owned facilities that are legal or social entities created for the purpose of producing goods and services, whose status does not permit them to be a source of income, profit or other financial gains for the unit(s) that establish, control or finance them; and, for-profit privately owned facilities that are legal entities set up for the purpose of producing goods and services and are capable of generating a profit or other financial gains for their owners.^27^

### Statistical analysis

Patients with and without VAP were compared using multiple logistic regression. Statistically significant variables were independently associated with an increased risk for VAP. The test statistic used was the Wald test, and the statistical significance level was set at 0.05. Calculated from the outputs of multiple logistic regression, adjusted odds ratios (aORs) and the corresponding 95% confidence intervals (CIs) of statistically significant variables were also reported.

We estimated variables independently associated with the outcome (VAP), adjusted to the following prospectively collected data: (1) sex (female or male), (2) age, (3) MV days before acquisition of VAP, (4) MV utilization ratio as a marker of severity of illness of patient, (5) type of respiratory support (continuous positive airway pressure [CPAP], endotracheal tube connected to a mechanical ventilator, tracheostomy connected to a mechanical ventilator, tracheostomy without connection to a mechanical ventilator, (6) hospitalization type (medical or surgical), (7) LOS, (8) ICU type (medical-surgical, medical, pediatric, surgical, coronary, neurosurgical, cardiothoracic, neurologic, trauma, pediatric oncology, or adult oncology), (9) facility ownership (publicly owned facility, not-for-profit privately owned facility, for-profit privately owned facility, or university hospital),^27^ and (10) income per country according to the World Bank classification (ie, low, lower-middle, upper-middle, or high).^25^

The evaluated outcome was the acquisition of VAP according to the CDC-NHSN definitions. All statistical analyses were performed using R version 4.1.3 software (R Foundation for Statistical Computing, Vienna, Austria).

To estimate VAP rates per country and per continent, we used the full database. To estimate risk factors for VAP, we included only those patients with data available for sex, age, and MV utilization ratio.

## Results

A cohort, prospective, multicenter, surveillance study of VAP was conducted in 743 ICUs of 282 hospitals in 144 cities in 42 countries from Asia, Africa, Europe, Latin America, and Middle East currently participating in the INICC: Argentina, Bahrain, Brazil, Bulgaria, China, Colombia, Costa Rica, Cuba, Dominican Republic, Ecuador, Egypt, El Salvador, Greece, India, Jordan, Kingdom of Saudi Arabia, Kosovo, Kuwait, Lebanon, Malaysia, Mexico, Mongolia, Morocco, Nepal, Pakistan, Palestine, Panama, Papua New Guinea, Peru, Philippines, Poland, Romania, Russia, Slovakia, Serbia, Sri Lanka, Sudan, Thailand, Tunisia, Turkey, United Arab Emirates, and Vietnam.

In this is a cohort study, the length of participation by hospitals ranged from 1.17 to 226.07 months (mean, 38.47; SD 42.62). Between July 1, 1998, and February 12, 2022, over 24 years, 289,643 patients admitted to 743 ICUs were followed for 1,951,405 patient days, and these patients acquired 8,236 VAPs.

**Fig. 1.**
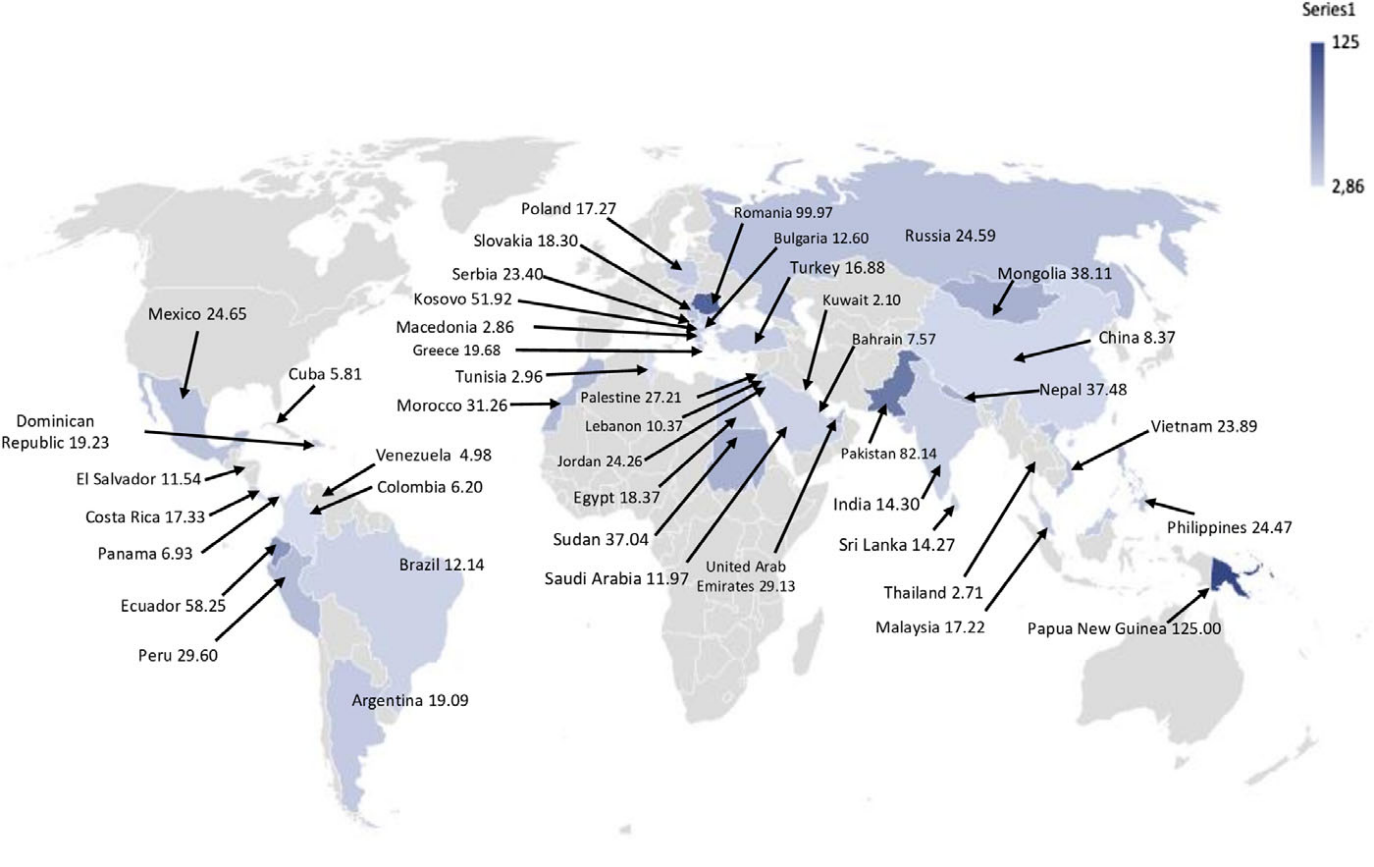
Rate of ventilator-associated pneumonia per 1,000 mechanical ventilator day, stratified per country.

Table 1 shows data on facility ownership, ICU type, and other participating hospital and patient characteristics. Rates of VAP stratified per country and per region are shown in Table 2 and Figure 1. VAP rates stratified per ICU type and per type of respiratory support are shown in Table 3. VAP rates stratified per World Bank country classification by income level (lower–middle income, upper–middle income, and high income) and by facility ownership type (publicly owned facilities, for-profit privately owned facilities, Teaching hospitals, and not-for-profit privately owned facilities) are shown in Table 3.

**Table 1.** Setting and Patient Characteristics, July 1, 1998, to February 12, 2022

Variable	Total
Study years, no.	24
ICUs, no.	743
Hospitals, no.	282
Cities, no.	144
Countries, no.	42
Total patients, no.	289,643
Total patients days, no.	1,951,405
Average LOS, mean (SD)	6.74 (8.33)
VAP cases, no.	8,236
**Survival status, no. (%)**	
Alive	249,461 (86.13)
Death	40,182 (13.87)
**Countries, stratified per income level according to the World Bank, no. (%)**	
Lower–middle-income country	11 (30.56)
Upper–middle-income country	19 (52.78)
High-income country	6 (16.67)
**Patients admitted per facility ownership, no. (%)**	
Publicly owned facilities	68,437 (23.63)
For-profit, privately owned facilities	121,792 (42.05)
Teaching hospitals	87,030 (30.05)
Not-for-profit, privately owned facilities	12,384 (4.27)
**Patients per hospitalization type, no. (%)**	
Medical hospitalization	210,427 (72.65)
Surgical hospitalization	79,216 (27.35)
**Patients admitted per type of ICU, no. (%)**	
Medical-surgical ICU	174,396 (60.21)
Medical ICU	32,212 (11.12)
Coronary ICU	26,940 (9.30)
Pediatric ICU	15,851 (5.47)
Surgical ICU	15,437 (5.33)
Cardiothoracic ICU	8,215 (2.84)
Neurosurgical ICU	5,710 (1.97)
Adult oncology ICU	3,573 (1.23)
Trauma ICU	2,724 (0.94)
Neurologic ICU	1,703 (0.59)
Pediatric oncology ICU	1,501 (0.52)
Respiratory ICU	1,381 (0.48)
**Sex, no. (%)**	
Male	176,382 (60.90)
Female	113,261 (39.10)
Age, mean (SD)	52.14 (23.93)
**Device days and device utilization ratio**	
MV utilization ratio, mean (SD)	0.27 (0.65)
Total MV days, no.; mean (SD)	702,335; 2.42 (6.19)
**Days using following types of respiratory support**	
CPAP connected to a MV	2,361
Endotracheal tube connected to a MV	93,574
Tracheostomy connected to a MV	3,068
Tracheostomy without connection to MV	719

Note. ICU, intensive care unit; MV, mechanical ventilator; LOS, length of stay; VAP, ventilator-associated pneumonia; SD, standard deviation; CPAP, continuous positive airway pressure.

**Table 2.** Ventilator-Associated Pneumonia Rates Stratified per Country and per Region

Country	Patients, No.	Patient Days, No.	VAP Cases, No.	MV Days, No.	VAP Rate, No.	95% CI
Argentina	23,590	168,234	829	43,431	19.09	19.04–19.12
Bahrain	1,224	11,205	48	6,338	7.57	7.50–7.64
Brazil	16,895	150,181	860	70,833	12.14	12.11–12.16
Bulgaria	992	9,544	79	6,272	12.60	12.50–12.68
China	4,324	35,999	99	11,827	8.37	8.31–8.42
Colombia	17,160	127,842	338	54,559	6.20	6.17–6.21
Costa Rica	1,469	6,413	37	2,135	17.33	17.15–17.50
Dominican Republic	1,418	10,569	52	2,704	19.23	19.06–19.39
Ecuador	944	16,826	250	4,292	58.25	58.02–58.47
Egypt	5,751	66,521	357	19,437	18.37	18.30–18.42
El Salvador	1,128	9,811	85	7,367	11.54	11.46–11.61
Greece	100	801	17	864	19.68	19.38–19.97
India	151,486	1,963,884	3,863	270,233	14.30	14.28–14.31
Jordan	5,106	39,200	244	10,058	24.26	24.16–24.35
Kosovo	248	3,462	58	1,117	51.92	51.50–52.34
Kuwait	7,047	101,688	85	40,409	2.10	2.08–2.11
Lebanon	6,292	54,448	210	20,244	10.37	10.32–10.41
Macedonia	3,550	21,939	28	9,783	2.86	2.82–2.89
Malaysia	5,748	43,913	468	26,409	17.72	17.67–17.77
Mexico	9,002	69,880	1,014	41,141	24.65	24.60–24.69
Mongolia	2,458	23,363	172	4,513	38.11	37.93–38.29
Morocco	3,583	25,061	225	7,197	31.26	31.13–31.39
Nepal	2,009	25,806	177	4,723	37.48	37.30–37.65
Pakistan	714	5,738	126	1,534	82.14	81.68–82.59
Palestine	1,264	64,988	123	4,521	27.21	27.05-27.35
Panama	948	8,771	48	6,929	6.93	6.86–6.98
Papua New Guinea	17	106	1	8	125.00	117.37–132.99
Peru	2,034	12,752	180	6,081	29.60	29.46–29.73
Philippines	5,480	33,028	392	16,055	24.42	24.34–24.49
Poland	1,908	23,011	264	15,287	17.27	17.20–17.33
Romania	977	8,465	397	3,971	99.97	99.66–100.28
Russia	98	1,116	3	122	24.59	23.71–25.48
Saudi Arabia	27,276	322,683	1,909	159,500	11.97	11.95–11.99
Serbia	186	1,862	15	641	23.40	23.02–23.77
Slovakia	938	9,293	107	5,846	18.30	18.19–18.41
Sri Lanka	327	2,398	14	981	14.27	14.03–14.51
Sudan	69	434	1	27	37.04	34.77–39.40
Thailand	649	2,774	2	739	2.71	2.58–2.82
Tunisia	221	1,909	4	1,351	2.96	2.86–3.05
Turkey	13,172	258,098	1,731	102,569	16.88	16.85–16.90
United Arab Emirates	385	53,273	6	206	29.13	28.39–29.87
Vietnam	4,280	51,644	442	18,504	23.89	23.81–23.95
**Region**						
Latin America	74,578	581,279	3,683	239,472	15.38	15.36–15.40
Asia	177,155	2,186,255	5,732	354,545	16.17	16.15–16.18
Eastern Europe	8,988	79,493	959	43,903	21.84	21.80–21.88
Middle East	70,115	934,520	4,809	367,336	13.09	13.08–13.10

Note. MV, mechanical ventilator; VAP, ventilator-associated pneumonia; CI, confidence interval.

**Table 3.** Ventilator-Associated Pneumonia Rates Stratified per ICU Type, per Type of Respiratory Support, per World Bank Country Classifications by Income Level, and per Facility Ownership Type

Variable	Patients, No.	Patient Days, No.	VAP, No.	MV Days, No.	VAP Rate	95% CI
**ICU type** ^**a**^						
Adult-oncology	1,381	13,438	92	9,679	24.96	24.84–25.07
Neurologic	1,703	11,702	52	3,389	15.34	15.21–15.47
Medical	5,710	38,669	121	11,806	14.07	14.04–14.10
Cardiothoracic	174,396	1,181,406	5,790	477,062	12.50	12.44–12.54
Pediatric oncology	3,573	17,748	173	6,931	12.23	12.05–12.40
Medical-surgical	32,212	234,303	911	64,731	12.14	12.13–12.15
Neurosurgical	8,215	49,858	225	18,004	10.25	10.19–10.30
Respiratory	2,724	13,357	27	4,593	9.51	9.44–9.56
Surgical	15,851	124,703	388	48,049	8.23	8.19–8.25
Pediatric	26,940	154,734	209	28,696	8.08	8.04–8.10
Coronary	1,501	9,288	19	1,554	7.28	7.25–7.31
Trauma	15,437	102,199	229	27,841	5.88	5.80–5.94
Pooled	289,643	1,951,405	8,236	702,335	11.73	11.72–11.73
**Respiratory support type**						
CPAP connected to a MV	2,361	18,187	252	4,092	61.58	61.34–61.82
Tracheostomy connected to MV	3,068	41,098	329	30,751	10.70	10.66–10.73
Endotracheal tube connected to MV	93,574	834,256	5,857	587,815	9.96	9.95–9.97
**Lower–middle income**						
Pooled	154,646	907,515	3,453	256,999	13.44	13.42–13.45
Publicly owned facilities	14,333	91,176	606	33,562	18.06	18.01–18.10
For-profit privately owned facilities	76,555	442,987	1,996	121,341	16.45	16.43–16.47
Teaching hospitals	52,805	312,669	707	86,626	8.16	8.14–8.18
Not-for-profit, privately owned facilities	10,953	60,683	144	15,470	9.31	9.26–9.35
**Upper–middle income**						
Pooled	98,839	699,513	3,277	273,755	11.97	11.96–11.98
Publicly owned facilities	22,515	167,783	963	75,883	12.69	12.66–12.71
For-profit, privately owned facilities	42,536	262,694	854	73,329	11.64	11.62–11.67
University hospitals	32,357	258,542	1,396	119,806	11.65	11.63–11.67
Not-for-profit, privately owned facilities	1,431	10,494	64	4,737	13.51	13.40–13.61
**High income**						
Pooled	36,158	344,377	1,506	171,581	8.78	8.76–8.79
Publicly owned facilities	31,589	300,701	1,208	148,744	8.12	8.10–8.13
For-profit, privately owned facilities	2,701	26,083	133	10,358	12.84	12.77–12.91
University hospitals	1,868	17,593	165	12,479	13.22	13.15–13.28

Note. ICU, intensive care unit; CI, confidence interval; MV, mechanical ventilator; VAP, ventilator-associated pneumonia; CPAP, continuous positive airway pressure.

^a^ICUs are listed in order of the highest to lowest ventilator-associated pneumonia rate.

Using multiple logistic regression, the following 6 variables were identified as statistically significantly independently associated with VAP: male sex; longer LOS, which increased the risk by 7% per day; MV utilization ratio; CPAP, which was associated with the highest risk; tracheostomy connected to a MV and endotracheal tube connected to a MV, which had the next-highest risk; surgical hospitalization instead of medical; public hospital; middle-income country; adult oncology ICU, which was associated with the highest risk; neurologic ICU and respiratory ICU, which had the next-highest risk. (Table 4). Coronary ICU showed the lowest risk.

**Table 4.** Multiple Logistic Regression Analysis of Risk Factors for Ventilator-Associated Pneumonia

Variable	aOR	95% CI	*P* Value
Age, y	1	1.00–1.01	.01
Sex, male	1.22	1.16–1.28	<.0001
Length of stay, d	1.07	1.07–1.08	<.0001
MV days	0.96	0.95–0.96	<.0001
MV utilization ratio	1.27	1.23–1.31	<.0001
**MV type**			
CPAP connected to a MV	13.38	11.57–15.48	<.0001
Tracheostomy connected to a MV	8.31	7.21–9.58	<.0001
Endotracheal tube connected to a MV	6.76	6.34–7.21	<.0001
Tracheostomy not connected to a MV	4.48	3.19–6.28	<.0001
**Surgical hospitalization**	1.23	1.17–1.29	<.0001
**Hospital type**			
Publicly owned facilities	1.59	1.35–1.86	<.0001
For-profit privately owned facilities	1.36	1.17–1.59	<.0001
Teaching hospitals	1.05	0.91–1.24	.48
**Country classification**			
Upper middle income country	1.22	1.15–1.29	<.0001
High income country	0.79	0.73–0.86	<.0001
**ICU type**			
Adult oncology ICU	4.05	3.22–5.09	<.0001
Neurologic ICU	2.48	1.78–3.45	<.0001
Respiratory ICU	2.35	1.79–3.07	<.0001
Medical-surgical ICU	2.15	1.85–2.49	<.0001
Medical ICU	1.99	1.68–2.34	<.0001
Neurosurgical ICU	1.26	0.98–1.63	.07
Pediatric ICU	1.19	0.97–1.43	.08
Pediatric oncology ICU	1.09	0.64–1.83	.76
Surgical ICU	1.02	0.83–1.24	.87
Trauma ICU	0.91	0.59–1.39	.67
Coronary ICU	0.63	0.51–0.77	<.0001

Note. ICU, intensive care unit; MV, mechanical ventilation; LOS, length of stay; VAP, ventilator-associated pneumonia; CPAP, continuous positive airway pressure; aOR, adjusted odds ratio; CI, confidence interval.

## Discussion

The VAP rates in the present study per country and per continent are significantly higher than those of the CDC-NHSN.^8^ This finding has been reported by the INICC since 2006^1^ and beyond.^2–8^

In the present study, we identified an association between male sex and VAP. In 1997, Kollef et al^28^ conducted a prospective cohort study in ICUs of Barnes-Jewish Hospital; they analyzed 521 ICU patients requiring MV for >12 hours. With multiple logistic regression analysis, they demonstrated that male sex was independently associated with the development of VAP.^28^

We further identified an association between the MV utilization ratio and VAP. In 2000, Sofianou et al^14^ conducted a prospective study to determine risk factors for VAP in 198 patients requiring MV for >48 hours. They found that MV for >10 days was a risk factor for VAP (OR, 44.4; 95% CI, 2.16–26.7; *P* < .0001).^14^

In our study, CPAP was associated with risk of acquiring pneumonia. Strategies to prevent VAP published by the Society for Healthcare Epidemiology of America (SHEA)–Association for Professionals in Infection Control and Epidemiology (APIC)–Infectious Diseases Society of America (IDSA) include the recommendation of using high-flow nasal oxygen or noninvasive positive pressure ventilation based on high quality of evidence, but there is no recommendation to use CPAP.^29^ A nationwide study conducted in Taiwan analyzed the impact of CPAP as a pneumonia RF. During 10 years, they identified adult patients with sleep apnea from the Taiwan National Health Insurance Research Database. A control cohort without sleep apnea, matched for age, sex and comorbidities, was selected for comparison. Of the 34,100 patients (6,816 study patients and 27,284 matched controls), 2,757 (8.09%) had pneumonia during a mean follow-up period of 4.50 years, including 638 (9.36%) study patients and 2,119 (7.77%) controls. Kaplan–Meier analysis showed a higher incidence of pneumonia among patients with sleep apnea (log rank test, *P* < .001). After multivariate adjustment, patients with sleep apnea experienced a 1.20-fold (95% CI, 1.10–1.31) increase in incident pneumonia. The risk was even higher among patients who use CPAP.^30^

We did not find a difference in the risk of VAP associated with age. However, Jovanovic et al^31^ conducted a prospective study to identify VAP RF, and age was independently associated with late-onset VAP. Furthermore, the Jovanovic study reported an association between surgical hospitalization and VAP compared with medical hospitalization. In our study, the ICUs with the highest risk for VAP were adult oncology ICU, respiratory ICU, and neurology ICU. The coronary ICU showed the lowest risk of VAP. The MV utilization ratio, as a marker of severity of illness of patients, is the highest in these types of ICUs,^32^ which could explain why these ICUs are associated with the highest risk of VAP.

Moreover, we detected an association between the acquisition of VAP rates in public hospitals compared with Teaching hospitals. However, a study^33^ conducted in neonatal ICUs found that the VAP rate per 1,000 MV days at Teaching hospitals was 13.2 (95% CI, 11.5–15.0). At public hospitals, this rate was 4.9 (95% CI, 2.5–8.6), and at private hospitals, this rate was 2.4 (95% CI, 1.3–3.9). Compared with public hospitals, Teaching hospitals showed a higher risk for VAP (relative risk [RR], 2.69; 95% CI, 1.50–4.80; *P* = .0001).^33^ In a study^34^ conducted in pediatric ICUs, the VAP rate per 1,000 MV days at Teaching hospitals was 8.3 (95% CI, 7.3–9.3). At public hospitals this rate was 4.7 (95% CI, 3.9–5.7), and at private hospitals this rate was 3.5 (95% CI, 2.6–4.5).^34^ Compared with private or public hospitals, Teaching hospitals showed a highest risk for VAP.^34^

In our study, patients admitted to ICUs in upper–middle-income countries were at higher risk for VAP than those admitted to ICUs in high-income countries. This finding could be explained by the lower quality of healthcare programs in middle-income countries participating in this study. A previous study^33^ conducted in NICUs reported a VAP rate per 1,000 MV days at lower–middle-income countries of 11.8 (95% CI, 10.1–13.6). In upper–middle-income countries, this rate was 6.7 (95% CI, 5.2–8.5). Compared with upper–middle-income countries, lower–middle-income countries showed a higher risk for VAP (RR, 1.75; 95% CI, 1.32–2.32; *P* = .0001).^33^ Another study^34^ conducted in PICUs reported a VAP rate per 1,000 MV-days in lower–middle-income countries of 9.0 (95% CI, 7.5–10.6). In upper–middle-income countries, this rate was 5.4 (95% CI, 4.8–6.1). Compared with upper–middle-income countries, lower–middle-income countries showed a higher risk for VAP.^34^

According with the most recent and also previous INICC reports^1–8^ published from 2006 to 2021, VAP is the most prevalent HAI in LMICs, and VAP is associated with high mortality, extra LOS, costs, and high bacterial resistance.^1–8^ To save countless lives in LMICs, it is essential to act quickly to control and prevent VAP.^10^ To do so, we suggest first focusing on identifying an evidence-based set of VAP prevention recommendations, such as those of SHEA–APIC–IDSA.^29^ Given that these organizations have already identified a number of evidence-based strategies to prevent the acquisition of VAP, it is crucial to be aware of this set of recommendations.^29^ Second, it is also recommended to monitor healthcare worker compliance with this set of recommendations and to provide them with performance feedback. This strategy has been effective in reducing the very high rate of VAP in LMICs.^35–42^ Last but not least, we suggest focusing on risk factors that can be changed to prevent VAPs. Some VAP risk factors are unlikely to change, such as sex, medical or surgical hospitalization, ICU type, facility ownership, and the country’s economy. Based on the our findings, addressing the following risk factors has the highest chance to reduce VAP: reducing LOS, limiting the duration of mechanical ventilation, limiting the use of CPAP. In addition, we suggest following a set of evidence-based recommendations to prevent VAP such as those published by the SHEA–APIC–IDSA.^29^

This study had several strengths. We use a prospective cohort study design. We collected data prospectively using standardized forms with a checklist for diagnosis of VAP, an online platform with dropdown menus to select the options of type of devices used, criteria for VAP, and diagnosis of VAP, to avoid typos in collected data. We used an electronic system to avoid bias in data collection among denominators and VAPs. IPPs who collected the data were trained individually by our principal investigator. This study was conducted across 24 years in 42 countries. All hospitals worldwide are invited to join this surveillance and research network and to collect data on HAIs using the INICC online platform (ie, participation is free).

Our research also had several limitations. First, this study is not representative of all hospitals in the 42 participant countries because hospitals voluntarily join the INICC and use this surveillance system for free. Second, the hospitals that participate in our surveillance system likely have better-quality HAI surveillance and prevention programs. Thus, the HAI rates in our study may be lower than the HAI rates in other hospitals not participating in our research. Finally, participating hospitals have not collected data on disease severity scores and underlying diseases, but we collected mechanical ventilation utilization ratio as a marker of severity of illness of patients, and we adjusted the analysis to this independent variable.
